# An Interdisciplinary Tutorial: A Self-Healing Soft Finger with Embedded Sensor

**DOI:** 10.3390/s23020811

**Published:** 2023-01-10

**Authors:** Ellen Roels, Seppe Terryn, Pasquale Ferrentino, Joost Brancart, Guy Van Assche, Bram Vanderborght

**Affiliations:** 1 Brubotics and Imec, Vrije Universiteit Brussel, Pleinlaan 2, 1050 Brussels, Belgium; 2 Physical Chemistry and Polymer Science (FYSC), Vrije Universiteit Brussel, Pleinlaan 2, 1050 Brussels, Belgium

**Keywords:** soft robotics, self-healing polymers, tutorial, soft sensors

## Abstract

In the field of soft robotics, knowledge of material science is becoming more and more important. However, many researchers have a background in only one of both domains. To aid the understanding of the other domain, this tutorial describes the complete process from polymer synthesis over fabrication to testing of a soft finger. Enough background is provided during the tutorial such that researchers from both fields can understand and sharpen their knowledge. Self-healing polymers are used in this tutorial, showing that these polymers that were once a specialty, have become accessible for broader use. The use of self-healing polymers allows soft robots to recover from fatal damage, as shown in this tutorial, which increases their lifespan significantly.

## 1. Introduction

Soft robotics is a fast-growing field that is getting more and more traction both in academia and in industry. The use of these robots has advantages in terms of safety and adaptability in unknown environments. To mature the field, reproducibility is an important topic to tackle. The development of competitions, open platforms and benchmarks is therefore important [1,2].

One of the trends in the field is to incorporate more and more material knowledge, and to use smart materials [3]. This sparks big opportunities for interdisciplinary research between robotics and materials science, but (graduate) students and researchers must acquire a sufficiently thorough understanding of the field that is not their speciality. This poses additional educational challenges that can be solved via workshops or tutorials [4].

To aid soft robotics researchers who wish to expand their knowledge, we developed a tutorial spanning different aspects of how to build your own self-healing soft robotics finger with embedded sensor. We discuss the synthesis of the polymer, the processing of the material in a soft robotic finger, and the sensor readout. This workshop was validated during the 1st International Winter School on Smart Materials for Soft Robots (12–17 December 2021) and was attended by 20 PhD researchers covering both fields. We follow the ambition of the Soft Robotics Toolkit [5] to have an open access repository on soft robotic parts and systems, hence all required materials are made available.

**Figure 1:**
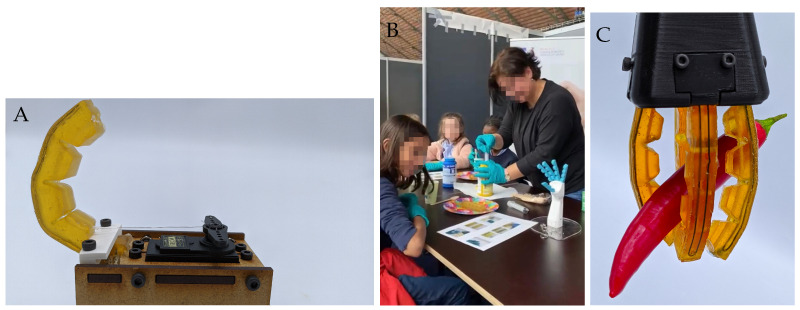
**Result of the tutorial and workshop.** (**A**): The self-healing soft finger with embedded sensor complete with the test set-up is the end goal of this tutorial. (**B**): The tutorial is easily adapted for use with commercial polymers, and can even be taught to children in an adapted version. No sensor was embedded in this case, and actuation was done manually. (**C**): The fingers made during the tutorial can for example be used in a gripper to pick up fruits.

This tutorial has been constructed around the use of a self-healing polymer containing thermo-reversible Diels-Alder (DA) bonds to manufacture a self-healing soft finger with embedded strain sensor (Figure 1). Such a finger can for example be used in a soft hand or a gripper. Although based on commercially available chemicals [6], these polymer materials are currently not commercially available, and interested readers are invited to contact the authors if they wish to obtain them. Yet, the concept of the tutorial is independent of the choice of the materials, although processing conditions, for example, would have to be adjusted. This has been proven during a workshop for children (targeted at primary school students, but all ages were welcome), where 150 children made their own soft finger. They did not use the self-healing materials, but a commercial silicone (Mold Star 16 Fast, Smooth-On).

In fact, many commercial materials can be used, such as the two-component silicone materials from Smooth-On that are often used in the field and well characterized [7,8]. They are generally safe, easy to work with, and thus recommended for people taking their first steps in soft robotics, but are not self-healing.

These silicones are thermosetting elastomers, meaning that the cross-links in the polymer matrix are chemical and irreversible. Because these cross-links are permanent, thermosetting elastomers do not melt and have a high temperature resistance. A disadvantage of this property is that they are not recyclable. Thermoplastic elastomers, the second category of elastomers, can be recycled. They are co-polymers consisting of soft blocks of polymer chains, with physical cross-links. These cross-links are physical and consist of hard blocks of polymer chains that are either glassy or crystalline. They are also thermally reversible and can be reformed using heat. When heated, thermoplastic elastomers become a viscous liquid. These are the materials typically used in fused filament fabrication 3D-printers, and can also be used for soft robotics.

The self-healing elastomers used during this workshop are a separate category, in the sense that they consist of chemical cross-links that are thermally reversible. The ability to recover their performance (actuation, sensing …) after fatal damage is an evident advantage of self-healing polymers for soft robotics. When healing these self-healing Diels-Alder networks, covalent (chemical) bonds are formed across the damage location, recovering the mechanical properties of the material. As such, self-healing prolongs the lifetime of the soft parts, reducing the amount of waste. This is an important advantage as sustainability becomes more and more important, with many researchers in soft robotics working on improving this aspect of the field [9,10]. Moreover, the advantages of these materials extend beyond this healing ability. The same mechanism can also be used to fuse parts together. Thanks to the covalent bonds that are formed, a strong interface is created. This greatly reduces the risk of delamination of the two parts, which is a common problem in soft robotics [11]. Moreover, strong interfaces can not only be formed between parts made out of the same self-healing material: When using different self-healing materials based on the same reversible chemistry, strong interfaces can also be achieved [12]. In this tutorial, this is illustrated at the interface between a self-healing sensor and the finger in which it is embedded.

Embedding a sensing ability in soft fingers is an important topic in soft robotics. As soft robots have essentially infinite degrees of freedom, controlling them can be challenging and is thus often done using artificial intelligence [13]. Embedding sensors enables to know the state of the robot and make control easier. Also, the location and morphology of the sensors is an important design decision [14]. This tutorial shows step-by-step how to embed a resistive strain sensor that is capable of estimating the bending of the finger.

**Figure 2:**
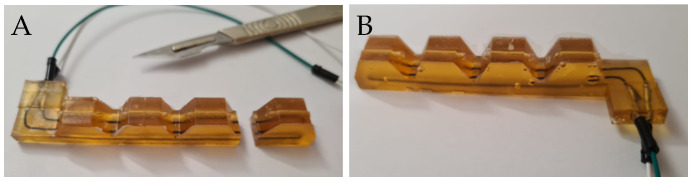
**Healing of the robotic finger.** The self-healing robotic finger that is developed during this workshop, can heal after being cut in two. It is tendon-driven and has an embedded resistive strain sensor to measure bending. (**A**): Finger cut in two. (**B**): Healed finger with recovered properties.

The tutorial describes the development of a tendon-driven self-healing soft finger with an embedded strain sensor, as shown in Figure 2, and is divided in four main parts: synthesis of the self-healing polymer, fabrication of the soft finger with embedded sensor, testing the robotic system, and damaging and healing the finger. The complete description of each step can be found in a designated Zenodo repository (DOI: 10.5281/zenodo.6581164 [15]), together with the required software and CAD files. The control aspect of soft robots is not covered in this tutorial, but interested readers are referred to review papers [13,16]. This article serves as a guide describing the reasoning behind the different steps, possible difficulties, pitfalls, and how to avoid them. Section 4 of this article evaluates the physical workshop and discusses the feedback from the participants.

## 2. Tutorial Description

The vision of this set-up is to make it low cost, and easily manufacturable using tools commonly found in a Fablab or maker space, such as a 3D printer and a laser cutter. Besides reagents and hardware, such as an Arduino nano, certain tools are required, a list of which is given in Table 1, together with a bill of materials for the tutorial as used during the physical workshop.

### 2.1. Synthesis of the Self-Healing Polymers

The self-healing behaviour of the polymers used during the workshop are based on thermally reversible Diels-Alder bonds. The Diels-Alder reaction used here is a chemical reaction between furan and maleimide groups. This reaction is an equilibrium reaction and can take place in both directions, either forming the Diels-Alder bonds, or breaking them, hence the reversibility. Both reactions occur simultaneously, though with different rates, eventually reaching an equilibrium where both reactions occur at the same rate. Therefore, the chemical equilibrium is called a dynamic equilibrium. At equilibrium at a given temperature, a fraction of the Diels-Alder bonds are formed, which is called the ‘equilibrium conversion’ *x_eq_*.

**Figure 3:**
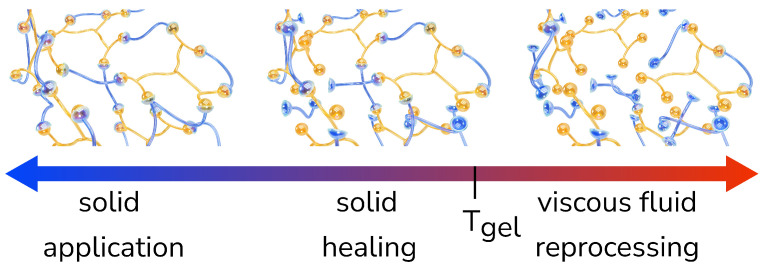
**The self-healing polymer forms a reversible network.** At room temperature, the polymer is solid and can be used for the application. It remains solid when heated mildly, which enables it to heal. Only when heated above its *T_gel_*, the polymer becomes a viscous fluid. Copyright 2022, Ellen Roels.

The kinetics (reaction rate) of both the forward and reverse reaction depend on temperature (via the Arrhenius equation). At low temperatures, both reactions are slow, and the equilibrium is such that most Diels-Alder bonds are formed, the equilibrium conversion approached 95% or more, resulting in a polymer network. With the increase of temperature, both reaction rates increase and the equilibrium shifts toward breaking more bonds and hence a decreasing equilibrium conversion (see Figure 3). This process continues with increasing temperature until eventually a point is reached where the macroscopic network no longer exists, having been broken down into smaller macromolecules. The temperature at which this happens is called the gel transition temperature *T_gel_*. Below this temperature, the material is a solid polymer, a macroscopic network held together by Diels-Alder bonds, while it becomes a viscous liquid when heated above *T_gel_*. Heating to even higher temperatures (>125 °C) should be avoided, as irreversible side reactions such as Michael additions and maleimide homopolymerization will start to take place. These side reactions have a negative influence on the self-healing ability of the material and should hence be avoided. To reduce the chance of side reactions, a radical inhibitor (4-tert-butylcatechol, 4TBC) is added to the polymer during the synthesis.

The self-healing process of the Diels-Alder polymers should be performed below *T_gel_*, such that it retains it structural stability during the procedure. The healing procedure is usually performed at temperatures just below *T_gel_*, to speed up the reaction kinetics and healing rate, as more Diels-Alder bonds are broken, also at the damage location. When gradually cooling down, these bonds are re-formed, including across the damage interface. Thus, healing at raised temperatures increases the healing efficiency. After waiting until the polymer reaches its equilibrium state at ambient temperature (usually 24 h is used), the mechanical properties are recovered, and the material is healed. Note that even at ambient temperature, the Diels-Alder reaction is taking place. While the material can theoretically heal in these conditions, the reaction rate is usually so low that it would typically take several months to fully recover the strength across the interface. By adapting the network design, materials can be synthesized that can heal at ambient temperature at a faster rate [17]. However, these room-temperature self-healing polymers have lower initial mechanical properties and healing may occur before broken parts are correctly re-assembled.

In this tutorial, two different self-healing materials are used. The matrix (BMI1400-FT3000-r0.5) is used for the soft finger, while the carbon black filled conductive composite (DPBM-FT5000-r0.6 + 20 wt% CB260 + 1 wt% C15A) is used for the embedded sensor. Since both materials are based on the same Diels-Alder chemistry, they can form bonds between them to create a strong interface [12].

#### 2.1.1. Conductive Composite

To make the self-healing polymer electrically conductive, carbon black is added during the synthesis. This approach is quite commonly used for making commercially available polymers, which are in general electrically insulating, electrically conductive, as carbon black is widely available [18,19]. Other approaches to make polymers electrically conductive include the addition of carbon nanotubes (CNT) [20], graphene [21], silver nanoparticles [22] or nanowires [23], …but safe-handling these nanofillers requires stringent precautions, as they pose mainly pneumological health risks, similar to those of asbestos. When working with these products, as with all chemical substances, it is important to study the safety datasheet in advance.

When adding carbon black to a polymer, the conductivity does not change linearly with the wt% of carbon black added. Instead, it follows a sigmoidal curve. The inflection point of this curve is called the ‘percolation threshold’, and at this point, the conductivity increases with several orders of magnitude for a small change in carbon black content. At a too low filler loading, no conductive pathway is present inside the material, and the conductivity is very low, at an insulating level. At sufficiently high filler loading, many conductive pathways form a percolating filler network, and thus the conductivity is high. Adding more filler would no longer change the conductivity significantly, as the electrical current finds already many paths to flow. When preparing a conductive composite for resistive strain sensors, the highest sensitivity is expected for a composition just above this percolation threshold: when straining such a material, the resistivity of the conductive particle network is affected the most. Another advantage of working just above the percolation threshold, is to limit the influence of the fillers on the mechanical properties of the material, as adding carbon-based fillers also has a stiffening effect, lowering the polymer chain mobility and the self-healing capacity [12]. The amount of carbon black needed for percolation, depends on the type and shape of the carbon black particles.

#### 2.1.2. Matrix Polymer

This tutorial was a great motivation to improve the broad applicability of the Diels-Alder polymers. Whereas a crystalline maleimide (DPBM) was used for the composite and most of our previous work [6,12], liquid bismaleimide (BMI1400) is used for the neat polymer matrix discussed here. It enables an easier synthesis, just requiring mechanical stirring of the liquid components, and does not require the use of a solvent that needs to be extracted in a later step. The solvent-less synthesis is safer, easier, and more ecologically friendly. Nevertheless, the necessary personal protection equipment needs to be worn when handling the reagents.

**Figure 4:**
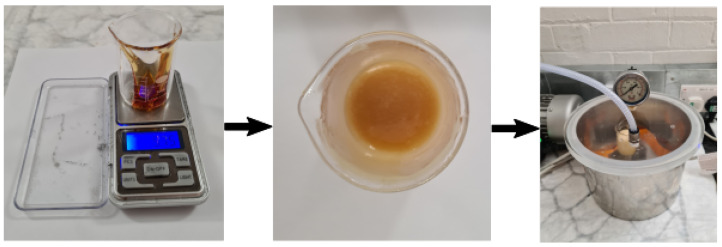
**Synthesis of the self-healing polymer.** First the correct amount of the different reagents (FT3000, BMI1400, 4TBC) has to be weighed (**left**). Afterwards, they need to be mixed well (**centre**). This creates trapped air bubbles, which are removed in a degassing step (**right**). A more detailed description of the synthesis can be found in the accompanying instruction document.

A stoichiometric ratio *r* of 0.5 between the maleimide and furan functional groups of the bismaleimide BMI1400 and the polyfuran resin FT3000 is used to obtain the desired soft elastomeric properties. The reagents need to be mixed well to obtain homogeneous properties. By vigorous stirring of the reagents, whether for the Diels-Alder polymer or for two-component silicone materials, air bubbles are introduced in the mixture (see Figure 4). When not addressed, these bubbles end up in the soft finger and create holes, and thus leaks, or influence the behaviour negatively. This often causes premature failure, especially in designs with thin structures, as is often the case in pneumatic soft robots. Trapped air bubbles can be removed by placing the stirred mixture in vacuum before further processing. In vacuum, the bubbles expand until they burst, releasing the air that was contained in them. Due to the foaming that takes place, a large container should be used (e.g., five times larger).

### 2.2. Fabrication of the Soft Sensorized Finger

There exist many different processing techniques to manufacture soft robots, and many of them can also be applied to self-healing polymers [24]. The use of these innovative materials has shown to have advantages over traditional polymers (thermosetting and thermoplastic), such as easier manufacturing of hollow structures and stronger multi-material parts.

Also for the Diels-Alder polymers used in this tutorial, different processing techniques have been explored [24].

The materials can be processed into a soft finger as part of the synthesis, where the liquid mixture cures inside a mould during the manufacturing, similar to what is done with thermosetting polymers. Alternative manufacturing techniques include solvent casting and casting [6].

The materials can also be thermally reprocessed after having been cured, something that cannot be done with conventional (irreversibly cross-linked) thermosetting polymers. For the Diels-Alder networks, this can be done by heating the material above its *T_gel_*, breaking bonds until the material starts to flow. At this point, the materials can be (re-)processed using approaches similar to thermoplastic materials. Techniques that have already been used include compression moulding, extrusion, fused filament fabrication (FFF), fused granulate fabrication (FGF), and selective laser sintering (SLS) [24]. But there is still an important difference: during this processing, a reaction is taking place, while this is not the case in thermoplastic polymers. Advantages of these self-healing polymers for additive manufacturing are that the final part is more isotropic, with strong covalent bonds connecting the different layers, easier to make airtight, and has smoother surfaces [24].

Moulding is used as manufacturing technique for the soft finger. Only easily accessible equipment is required for this technique, hence lowering the bar for using the self-healing materials, and taking the first steps into the field. Additionally, this makes this tutorial also easier to give as a workshop outside a (chemical) lab environment. The moulds that were used during the physical workshop were printed using a Prusa SL1, which is an SLA 3D-printer, and using 3DJake Color Mix resin. The designs can be found in the Zenodo repository [15]. When an SLA printer is not available, moulds can also be printed using FFF (widely available, but lower accuracy and higher surface roughness) or SLS (limited availability), or can be manufactured using a CNC machine (more expensive), out of cardboard (single use and limited design freedom) [25], or made by casting the mould in a stiffer silicone (shore hardness around 50A is recommended). In this case, a positive mould of the finger should be obtained.

While there have been many reports on curing inhibition of (platinum-cured) silicone materials when using SLA printed moulds, these difficulties have not yet been reported with Diels-Alder based polymers. The cure inhibition of silicone is a known problem that can be solved by washing the mould in isopropyl alcohol, post-curing it under UV light from different angles (either in a UV chamber or in sunlight for several hours), and baking it at 60 °C for several hours [26].

Release agent can be sprayed on the moulds to make the removal after curing easier, but is not required. When using release agent, the cured part should be washed to remove its residuals. The residual coating of the release agent has a negative impact on the healing of Diels-Alder polymers at the coated interfaces. Curing can be done at room temperature, or at a mild temperature (up to 90 °C in this case, but material-dependent). At raised temperatures, the reaction is accelerated and the curing is faster (1.5 h). After taking it out of the oven, the parts can only be demoulded after the mould is slowly cooled down to room temperature. At this point, the material is not yet at equilibrium, and is not at its full mechanical strength, which is not strictly necessary for demoulding.

The moulding procedure of the sensorized soft finger consists of two steps: first the sensor is moulded, and subsequently it is embedded in the mould of the finger and overmoulded. This requires the material of the first step to cure to an advanced conversion before starting the second step, otherwise dissolution could occur. Once the sensor is cured sufficiently, it is taken out of the mould and inserted in the mould of the soft finger for the overmoulding step.

#### 2.2.1. Sensor Fabrication

The sensor consists of a conductive composite fibre with a diameter of 0.5 mm. This fibre is manufactured using a piston extruder, the process is detailed in previous work. During the physical workshop, this conductive fibre was given to the participants. Making a good connection between soft and hard materials has been a difficult topic in soft robotics, as stress concentrations occur at the interfaces. Therefore, extra care has to be taken when making such a connection, for example between the soft sensor fibre and the copper wiring of the measurement circuit. After careful consideration, we found that the optimal way is to use female DuPont style crimp connectors that are crimped directly on the sensor fibre. These connectors are compatible with standard jumper wires, making a connection with readout electronics straightforward.

**Figure 5:**
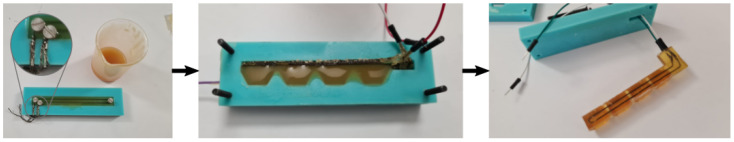
**Moulding the sensor and finger.** The mould for the sensorized finger has three bolts around which the sensor fibre is wrapped, and cutouts to keep the crimp connectors in place. The fibre is fixed, and the liquid polymer mixture is poured over it. After curing, the sensor is placed in the finger mould before the finger is cast. When the finger has cured, it can be demoulded and tested.

The easiest way to incorporate a strain sensor in a soft robotic finger, is a single straight line with the sensor sticking out at both ends. While easy to make, this is not practical in applications, where the electrical read-out should rather occur at the base of the finger, so no wires are dangling from the tip. This can be solved by making a U-shaped sensor, such that both ends are on the same side (the base). To keep the sensor fibre in a U-shaped position during the overmoulding and curing of the surrounding finger, the mould contains metal inserts or bolts, around which the fibre is wrapped (Figure 5). Cutouts in the mould keep the crimp connectors in place.

#### 2.2.2. Soft Finger Fabrication

Once the sensor is positioned, the liquid matrix mixture for the finger is poured over the sensor (see Figure 5) and cured at room temperature or, preferably, in an oven, as described before. By curing at a raised temperature in an oven, the curing process is faster and some of the bonds in the sensor fibre are broken, ensuring the full interfacial strength is obtained faster during cooling. After filling the mould, a lid is placed on the mould to obtain a uniform thickness and reduce the capillary effect. An opening is present in the lid for the crimp connectors, through which some extra material can be poured if needed. While pouring, care has to be taken to avoid forming extra bubbles. To reduce any remaining bubbles, the mould can be placed in a pressure chamber. Under higher pressure, the bubbles will be squeezed and have less influence on the final result.

Due to the nature of the self-healing materials, no glue is needed to obtain high interfacial strength and good load transfer between the sensor and the rest of the soft finger. Both parts are effectively fused together with reversible covalent bonds. This eliminates a common cause of failure of soft robots: the delamination of parts having a different stiffness. Additionally, any holes that remain after curing can be filled by heating up some previously cured material until it flows (for example using a soldering iron), and dripping or injecting it into the cavities. This makes the failure rate of the procedure very low, giving an advantage over many common materials used to fabricate soft robots.

When the finger is cured, it can be carefully taken out of the mould. To make it ready for testing, small pieces of PTFE tubing are inserted in the phalanges. The nylon tendon wire is routed through these PTFE tubes. These tubes protect the soft finger from being damaged by the tendon wire when this tendon is tensioned.

### 2.3. Testing the Robotic System

**Figure 6:**
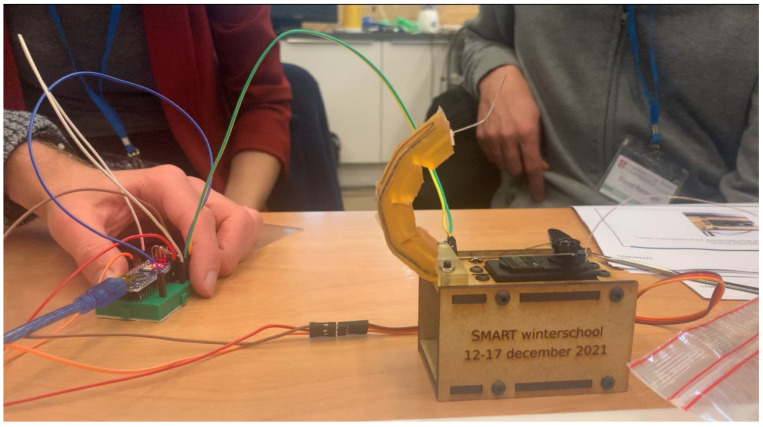
**Participants testing the set-up.** Participants successfully built the set-up and are actuating the finger while recording the strain sensor values on their computer.

The moulded finger can be tested by the participants on a small test set-up that was developed for its ease of fabrication and limited cost, while still being able to show the most important concepts. The frame consists of laser cut medium density fibreboard (MDF) bolted together, as shown in Figure 6. It holds a servo motor that is used to pull the tendon and make the finger bend. While a cheap servo motor like this does the job well, it can be swapped to a stepper motor or a high-end servomotor (for example Dynamixel) for more positional accuracy. To this end, the CAD drawing can easily be adapted.

The resistance of the sensor fibre is measured using a voltage divider. The sensor itself acts as one strain-dependent (variable) resistor, and a second (fixed) resistor is placed in series with it. The resistance of the sensor can be derived from a voltage measurement over the sensor itself using the formula:

$$R_{\mathrm{sensor}}=R_{\mathrm{fixed}}\frac{V_{\mathrm{out}}}{V_{\mathrm{in}}-V_{\mathrm{out}}}$$

where $V_{\mathrm{in}}$ is in this case 5 V, as this voltage is drawn from the Arduino. This leaves the question of how to choose the value of the fixed resistor $R_{\mathrm{fixed}}$. It should be chosen such that the difference in output voltage $V_{\mathrm{out}}$ when the finger is straight compared to when it is fully bent, is maximal. The maximal difference is achieved when the resistance is chosen as the geometric mean of the maximal and minimal resistance of the sensor, or $R_{\mathrm{fixed}}=\sqrt{R_{\mathrm{sensor},\min}\cdot R_{\mathrm{sensor},\max}}$. As in this case the resistance of the sensor $R_{\mathrm{sensor}}$ drops when bent, a fixed resistance in the same order of magnitude as the value of the sensor at rest, but slightly lower, is also a good pick.

The output voltage is measured by the Arduino nano’s internal analogue to digital converter (ADC), which has a 10-bit resolution. This means that the Arduino registers the voltage as a value between 0 (= 0 V) and 1024 (= 5 V), which has to be converted to have the actual input voltage $V_{\mathrm{in}}$, and thus the sensor resistance $R_{\mathrm{fixed}}$. If a more accurate measurement is desired, additional parts are required, such as an analogue-digital converter with a higher resolution or a Wheatstone bridge. Alternatively, a digital multimeter can be used that already contains these parts. Using such a device is a good option when working in a lab environment, however unpractical to integrate in a (soft) robot for further control purposes.

To calibrate the sensor, and get a relation between the bending angle of the finger and the resistance of the sensor, a camera can be used [27]. The bending angle is defined as the angle between the resting position of the finger and the line connecting base and tip of the finger. A camera is not included in the set-up, to limit the complexity of this tutorial. When adding a camera, or any other measurement device, it should use the same clock for all measured variables, or synchronize the different clocks using an event trigger. As it is more complex to add a camera to an Arduino, a computer such as the low-cost Raspberry Pi could be used instead. The images can be post-processed using tools like OpenCV, but this requires some programming experience. Alternatively, the test can be performed on a background of graph paper, which enables post-processing by hand. For more information about the calibration of these sensors, the reader is referred to our previous work [12].

### 2.4. Healing the Finger

As the last part, the healing of the finger is studied. First, dismount the finger from the setup and note the resistance of the sensor. Many types of damage can be considered. In this tutorial, fatal damage was considered as a demonstration of the healing ability of both actuator and sensor. After cutting the finger in two (perpendicular to its length is suggested), it needs to be realigned correctly. This realignment is done by positioning the pieces such that the resistance of the sensor fibre is again in the same order of magnitude as the original value. To make the realignment easier, sensor fibres of 0.5 mm diameter are used. This diameter was found to be a good compromise: thinner fibres would be more difficult to align correctly, while thicker fibres have a larger stiffening effect on the soft finger.

After verifying the alignment, the finger is placed in an oven at 90 °C for 45 min to heal. After healing, the finger should rest at room temperature to reach its equilibrium and recover the broken Diels-Alder bonds. 24 h later, the mechanical properties have mostly recovered [17,28]. For the intended actuation purpose, the finger does not need its full strength to function. The mechanical properties should be recovered to about the double of the typical stress and strain expected to undergo during normal actuation. Hence, it can be tested again sooner.

## 3. Characterization of the Robotic Finger

**Figure 7:**
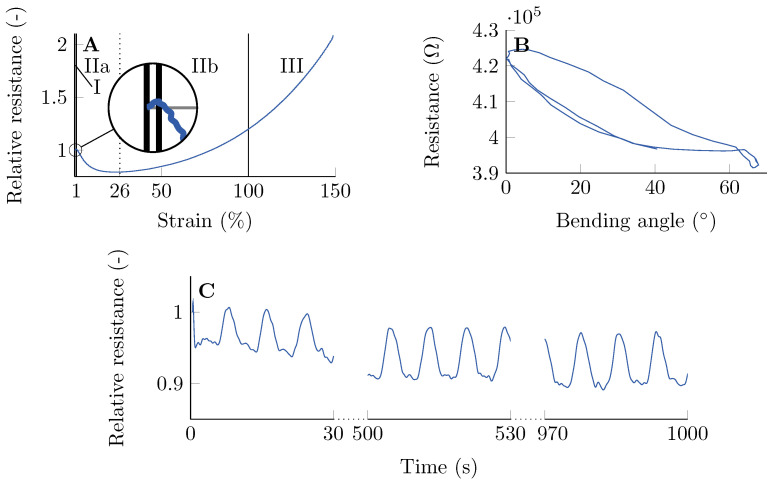
**Characterization of the sensor.** (**A**): Four zones can be distinguished in the fibre response. Zones I and IIa are the working domain of the sensor. Adapted from [12]. (**B**): The sensor embedded in the finger shows some hysteresis and reaches a maximal bending angle of 70${}^{{}^{\circ}}$. (**C**): The finger is cyclically subjected from its rest position to its maximal bending angle. The sensor shows a drift of 3% after 1000 s.

The self-healing finger developed during this tutorial is based on the one developed in our previous work [12]. An extensive characterization and rationale about the choice of the sensor material can be found there. When strained, the resistivity of the fibre follows the model that was first described by Flandin et al. [29] Four zones can be distinguished on the graph of Figure 7A. Zone I, the initiation zone, is only visible the first time the fibre is strained and is therefore not visible in Figure 7B,C. Zone II (reversible) is split in IIa and IIb at the point where the resistance starts to increase again (26 %). Zones I and IIa are the main operation zones of the sensor, as in this region, there is a one-on-one relationship between strain and resistance. Zones IIb and III (recoverable damage) are therefore not considered as the working domain of the fibre. During the test, the fibre failed before zone IV (depercolation, as described by Flandin et al.) was reached.

The finger with embedded sensor was subjected to a cyclic test (Figure 7B,C), during which it was repeatedly actuated up to its maximal bending angle. The maximal angle reached was 70${}^{{}^{\circ}}$, measured between the horizontal line and the line drawn from the base where it was clamped to the tip. When bending, the fibre is strained and the resistance decreases, corresponding to zone IIa. Some hysteresis and drift (3% after 1000 s) of the embedded sensor are noticeable, which are not uncommon for soft elastomeric sensors [18]. These properties make the reconstruction of the strain/bending from the resistance signal less straightforward, but can be solved using more extensive models or artificial intelligence [30].

## 4. Evaluation of the Physical Workshop

This tutorial was given physically to 20 PhD students with either a robotics or a materials science background, divided in five mixed groups, so they can learn better from each other during this workshop spanning both disciplines. Each group mounted a sensor fibre in a finger mould, prepared the reactive mixture, and cast it around the sensor. As the workshop duration was only 2.5 h, there was no time to wait for the material to cure. Therefore, the groups were given pre-made sensors and soft fingers to continue the tutorial. Within the allocated time, all groups had an operational actuator and completed the tutorial.

### 4.1. Participant Issues during the Workshop

During the workshop, some minor issues were recorded. One group accidentally damaged the connectors of the pre-made sensor, while another group damaged the connectors of the pre-made finger. This indicates that the connectors are one of the most fragile parts of the design. While the pieces that broke off can be healed again to repair the finger, it takes too long to do so during the workshop itself. These groups were handed a spare sensor/finger. A third group had some issues with a broken servo motor and jumper wire, they were also handed replacements.

A different issue was caused by the Arduino Nano used. An extra driver was required to be able to program it using the Arduino IDE, which was provided on a USB drive. Some groups were unable to program it using their own laptop, while reading the sensor data was not a problem. Their code was uploaded to the device using a different laptop.

### 4.2. Overall Experience

The workshop was followed by 20 PhD students, of which 17 filled in the optional questionnaire at the end. Of these 17, 10 participants were from the field of (soft) robotics, and 7 from materials science.

On the questionnaire, the participants were asked to rate several questions on a 1–10 point scale to get an overview of their prior knowledge, learning, and overall experience during the workshop. Most students (10) reported that they did not have much experience with self-healing polymers before this tutorial (score: ≤5 with 1: no experience, 10: highly experienced) of which 6 stating that they had no experience (score: 1). As for experience in soft robotics, only 4 students reported a score ≤5 (1: no experience, 10: highly experienced).

**Figure 8:**
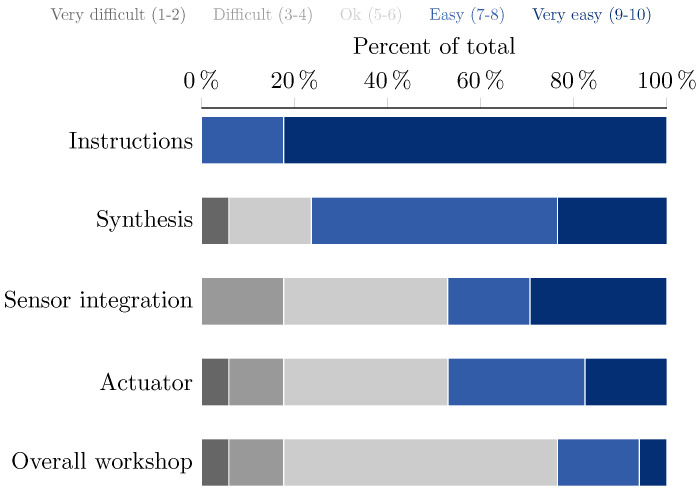
**Difficulty analysis** of the instructions, the workshop and its parts. The data is obtained from the questionnaire.

The participants were also asked to score the difficulty of the workshop and its parts: material synthesis, sensor integration, and the actuator. In addition, a score was given on the difficulty of the instructions given. The results of this difficulty analysis are shown in Figure 8. All participants found the instructions easy to follow, and the synthesis of the materials was also mostly perceived as not too difficult. This indicates that the goal of making the self-healing polymers easy to use has been reached. The overall workshop difficulty was rated as ‘ok’, which we believe is good as it is neither too hard, nor too difficult.

Overall, the students were highly satisfied with the quality of the hands-on workshop (9.5 ± 0.2, with 1: very poor, 10: excellent), and enjoyed it a lot (9.6 ± 0.2, with 1: I hated it, 10: I loved it).

## 5. Conclusions

This article focused on the scientific and technological principles of the sensorized self-healing soft robotics finger manufactured and tested during this tutorial. The detailed instructions for the tutorial are given in the Zenodo repository [15]. An introduction to the working principle of the self-healing Diels-Alder polymers and their processing techniques was given, and is also necessary for the students involved to better understand the underlying principles of the different steps and procedures used during the tutorial. After synthesizing and moulding the sensorized soft finger, it is tested on a dedicated but low-cost set-up, providing insights in the response of the strain sensor to the bending of the finger. The sensorized actuator was subjected to fatal damage by cutting the actuator into two pieces. The pieces were joined together to heal the damage and both actuation and sensing performance were recovered. To increase the usefulness of this tutorial, possible alternatives and extensions suggested are left to the reader to implement. The tutorial was validated during a physical workshop followed by 20 PhD students with different backgrounds, and was received positively.

## Figures and Tables

**Table 1:** List of tools and bill of materials. Components are sorted by the part in which they are first used. Components used in multiple parts are only listed once. For detailed info, see the instruction document on the Zenodo repository [15].

*Tools*	
□ Lab coat	□ Gloves
□ Ruler	□ Scale 0.01 g
□ Cutting board	□ Recipient
□ Scalpel	□ Spatula
□ Pliers	□ Cleaning paper
□ Multimeter	□ Vacuum chamberr
□ Flathead screwdriver	□ Oven
**Item**	**Amount**
***Synthesis***	
Bismaleimide BMI1400	6 g
Radical inhibitor 4TBC	0.06 g
Polyfuran resin FT3000	9 g ${}^{a}$
Acetone	a few mL
Sheet of paper	1
Pipette (plastic)	1
***Fabrication***	
Self-healing sensor fibre	18 cm ${}^{a}$
Jumper wires	9
Acrylic mould set (SLA printed)	1 ${}^{a}$
Various bolts & nuts	N/A
Release agent	a bit
Copper wire	30 cm
Crimp connectors	2
Nylon wire (diameter 0.5 mm)	30 cm
PTFE tubing	10 cm
***Testing the robotic system***	
Motor holder set (laser cut)	1 ${}^{a}$
Servo motor	1
Arduino nano + USB cable	1
Resistor	1
Breadboard	1

^a^ Made in-house.

## Data Availability

All data and supporting material can be found in the Zenodo repository [15].
